# Acetyl group coordinated progression through the catalytic cycle of an arylalkylamine N-acetyltransferase

**DOI:** 10.1371/journal.pone.0177270

**Published:** 2017-05-09

**Authors:** Adam A. Aboalroub, Ashleigh B. Bachman, Ziming Zhang, Dimitra Keramisanou, David J. Merkler, Ioannis Gelis

**Affiliations:** Department of Chemistry, University of South Florida, Tampa, Florida, United States of America; California State University Fresno, UNITED STATES

## Abstract

The transfer of an acetyl group from acetyl-CoA to an acceptor amine is a ubiquitous biochemical transformation catalyzed by Gcn5-related N-acetyltransferases (GNATs). Although it is established that the reaction proceeds through a sequential ordered mechanism, the role of the acetyl group in driving the ordered formation of binary and ternary complexes remains elusive. Herein, we show that CoA and acetyl-CoA alter the conformation of the substrate binding site of an arylalkylamine N-acetyltransferase (AANAT) to facilitate interaction with acceptor substrates. However, it is the presence of the acetyl group within the catalytic funnel that triggers high affinity binding. Acetyl group occupancy is relayed through a conserved salt bridge between the P-loop and the acceptor binding site, and is manifested as differential dynamics in the CoA and acetyl-CoA-bound states. The capacity of the acetyl group carried by an acceptor to promote its tight binding even in the absence of CoA, but also its mutually exclusive position to the acetyl group of acetyl-CoA underscore its importance in coordinating the progression of the catalytic cycle.

## Introduction

GNAT enzymes catalyze the acetylation of a wide range of primary amine acceptors from histone and ribosomal proteins, to small molecules like serotonin, dopamine and aminoglycoside antibiotics [1–3]. Thus, they are central regulators of diverse cellular processes such as gene transcription [4], time and seasonal adaptation [5–7] and metabolism [8, 9]. Structural analysis of GNAT family members guided primarily by crystallography has revealed a conserved core domain, made up of a mixed β sheet and flanking α helices, which contains four signature motifs (A-D) [1–3]. Strands β4 and β5, from motifs A and B, respectively, form the only parallel stretch of the mixed β sheet. A conserved β bulge in strand β4 results in partial separation of the β4- β5 stretch, creating a characteristic V-shaped catalytic cavity that interacts with the pantetheine arm and the acetyl group of acetyl-CoA. The amide nitrogen of the residue downstream of the β bulge forms a conserved hydrogen bond with the carbonyl of the acetyl group. In contrast to the pantetheine arm and the acetyl group which reach deep in the aforementioned cavity, the 3’-phosphate ADP is exposed at the surface of the protein, resulting in an overall bent conformation for acetyl-CoA. The highly conserved Q/R-X-X-G-X-G/A consensus sequence found at the loop downstream of β4 (P-loop) coordinates the pyrophosphate moiety of the 3’-phosphate ADP via hydrogen bonding of main-chain atoms. Although the mode of acetyl-CoA binding is highly conserved, the mode of interaction of GNATs with acceptor substrates is highly divergent due to their substantially different chemical nature [1–3].

Arylalkylamine N-acetyltransferases (AANATs) catalyze the transfer of an acetyl group to a diverse gamut of arylalkylamines, such as indolethylamines and phenylethylamines [10, 11]. In mammals, a single *AANAT* gene is expressed in the pineal gland and in the retina, where it is involved in the melatonin pathway [12], and retinal neurotransmission and detoxification [13, 14], respectively. AANAT, also known as serotonin N-acetyltransferase (SNAT), is the penultimate enzyme in the melatonin biosynthesis pathway and it catalyzes the N-acetylation of serotonin using acetyl-CoA as acetyl donor to produce N-acetylserotonin [15]. On the other hand, insects have multiple copies of *AANATs* that function beyond the regulation of the circadian system, particularly in neurotransmitter metabolism [16, 17] and sclerotization and pigmentation [18–21]. Structural studies on three AANATs identified in *Aedes aegypti*, revealed that the three enzymes share high structural homology and contain the signature motifs of the GNAT superfamily, but display distinct binding specificities with respect to the acceptor substrate [22]. Accumulating evidence from kinetic analyses of AANATs, has shown that they follow an ordered, ternary complex mechanism, where acetyl-CoA binding facilitates the interaction with the acceptors [15, 23–25]. Indeed, the structure of the prototypical AANAT, serotonin N-acetyltransferase, from *Ovies aries* (*oa*AANAT) in the free state and when in complex with a bisubstrate analog supports this mechanism, as the acceptor binding site is fully formed only after acetyl-CoA-induced conformational changes [26, 27]. These changes involve rearrangement of loops over the acetyl-CoA binding cavity as well as a notable loop-to-helix transition at the C-terminal end of helix α1. However, significant deviations from this model are observed. Comparison of the crystal structures of the highly homologous dopamine N-acetyltransferase from *Drosophila melanogaster* (DAT) solved in the free and acetyl-CoA-bound forms, revealed that the acetyl-CoA induced loop-to-helix transition at the C-terminal end of helix α1 observed for *oa*AANAT preexists in the free form of DAT [28]. Similarly, comparison of the crystal structure of free *Aedes aegypti* AANAT2 (*aa*AANAT2) to that of free or acetyl-CoA bound DAT shows the same distribution and three dimensional arrangement of secondary structure elements, suggesting that, again, the conformational changes observed for *oa*AANAT preexist in the free form of *aa*AANAT2 [22, 28]. Thus, even for closely related GNAT homologues as the AANAT subfamily members, a common, ternary complex kinetic mechanism can be driven by distinct underlying molecular events. In addition, more divergent models, such as global unfolded-to-folded transitions, are encountered when GNATs other than AANATs are considered [29, 30].

In the current study we used a combination of nuclear magnetic resonance spectroscopy (NMR), isothermal titration calorimetry (ITC) and homology modelling to follow the formation of binary and ternary complexes during the catalytic cycle of *bm*AANAT3, a small GNAT from *Bombyx mori* involved in the acetylation of a broad range of amines. Our data reveal that the acetyl group plays a central role in coordinating the ordered progression of AANATs through the binding and release events encountered during the ternary complex mechanism, by regulating the conformational properties of both, the donor and acceptor binding sites.

## Materials and methods

### Expression and purification of *bm*AANAT3

Full-length *bm*AANAT3 cloned into a pET28a vector (thrombin cleavage site and kanamycin resistance) was expressed in BL21(DE3) competent *E*. *Coli* cells (New England Biolabs) as a fusion protein with an N-terminal His-tag. Cell growth was performed at 37°C and protein expression was induced at an OD_600_ = 0.5 by the addition of 1 mM IPTG, for 5 hours. Cells were harvested by centrifugation at 4°C, resuspended in 50 mM Tris-HCl, pH = 8.0, 20 mM imidazole, 500 mM NaCl, 1 mM Phenylmethylsulfonyl fluoride and 5 mM β-mercaptoethanol and lysed by sonication. Cell debris were removed by centrifugation at 35,000 x g (4°C). The clarified lysate was loaded on a Ni^2+^ affinity column, washed with 10 column volumes of cell lysis buffer and eluted with 400 mM imidazole in the same buffer. Fusion *bm*AANAT3 was further purified through a Superdex-75 gel-filtration column using a 50 mM Tris-HCl pH = 8.0, 150 mM NaCl, 0.5 mM EDTA and 5 mM β-mercaptoethanol buffer. The purification polyhistidine tag was removed by incubating *bm*AANAT3 with biotinylated thrombin (2U/mg). ^15^N- or ^13^C/^15^N-labeled *bm*AANAT3 was prepared using the same expression and purification protocol, except that cells were grown in M9 media containing ^15^N-NH_4_Cl and ^13^C-glucose as the sole source of nitrogen and carbon, respectively.

### NMR spectroscopy

All NMR spectra were acquired in 20 mM Tris, 100 mM KCl, 5 mM DTT, 0.5 mM EDTA and 7% D_2_O, at 30°C, using Varian direct drive 600 and 800 MHz spectrometers equipped with a cryogenic probe. {^1^H}-^15^N NOE experiments were acquired in an interleaved manner and with a recycling delay of 5 s. Spectra were processed using NMRPipe [31] and analyzed using Sparky (T. D. Goddard and D. G. Kneller, SPARKY 3, University of California, San Francisco). *bm*AANAT3 titrations with acetyl-CoA and CoA were performed at 30°C and at a protein concentration of 250 μM. The titration with acetyl-CoA progressed to 750 μM. Two titrations were performed with CoA, one in small increaments that progressed up to 1.75 mM for extracting the K_d_ of the interaction, and one in large increments that progressed to 8 M and was used to achieve > 99.6% saturation. The assignment of free *bm*AANAT3 has been reported previously and can be found at the BMRB under the accession number 26962 [32]. The assignment of the ^15^N-HSQC of *bm*AANAT3 in the CoA-bound state was performed by following the signal trajectories during the course of the titration. For the assignment of the ^15^N-HSQC of *bm*AANAT3 in the acetyl-CoA-bound state, the spectrum of the CoA-bound state was used. Titrations with substrate acceptors in the presence or absence of acetyl-CoA or CoA were performed at 22°C at a concentration of 120–150 μM, and progressed close to saturation.

Combined chemical shift changes were calculated using the following expression:

$$\Delta \delta =\sqrt{\Delta \delta_{\text{H}}^{2}+{\left(\frac{\Delta \delta_{\text{N}}}{5}\right)}^{2}}$$

Dissociation constants were determined by global fitting of several residues to [33]:

$${\Delta \delta }_{\text{obs}}{=\Delta \delta }_{\text{max}}\frac{\left(\left[{\text{P}}_{\text{t}}\right]\text{+}\left[{\text{L}}_{\text{t}}\right]{\text{+K}}_{\text{d}}\right)-\sqrt{{\left(\left[{\text{P}}_{\text{t}}\right]\text{+}\left[{\text{L}}_{\text{t}}\right]{\text{+K}}_{\text{d}}\right)}^{2}-4\left[{\text{P}}_{\text{t}}\right]\left[{\text{L}}_{\text{t}}\right]}}{2\left[{\text{P}}_{\text{t}}\right]}$$

where Δδ_obs_ is the incremental chemical shift change at each point of the titration, Δδ_max_ is the maximum chemical shift change (at saturation), [P]_t_ and [L]_t_ are the total protein and ligand concentrations at each point of the titration and K_d_ is dissociation constant.

### Isothermal titration calorimetry

ITC experiments were performed on a VP-ITC calorimeter (GE Healthcare) at 30 and 22°C for cofactors and acceptor substrates, respectively. *bm*AANAT3 was dialyzed against 20 mM Tris, 100 mM NaCl, 0.5 mM EDTA and 1 mM tris(2-carboxyethyl)phosphine and degassed. *bm*AANAT3 concentration was ~40 μM and the ligands at a 10- to 100-fold excess. Titrations included an initial 0.2-μl injection and were completed by 10–12 injections, with a 4 min time interval between each injection. The data were processed using Origin 7.0 (OriginLab Corporation) with the point of the initial injection excluded and using an one-site binding model.

### Homology model

The structure of *bm*AANAT3 was generated with SWISS-MODEL [34] using the crystal structure of the arylalkylamine N-Acetyltransferase 2 from the yellow fever mosquito, Aedes aegypti (*aa*AANAT2, PDB: 4FD6) [22] as a template. *aa*AANAT2 is found in the same cluster of a phylogenetic tree of insect aaNATs as *bm*AANAT3 and exhibits 47% sequence identity and 62% sequence similarity to *bm*AANAT3.

## Results and discussion

**Fig 1:**
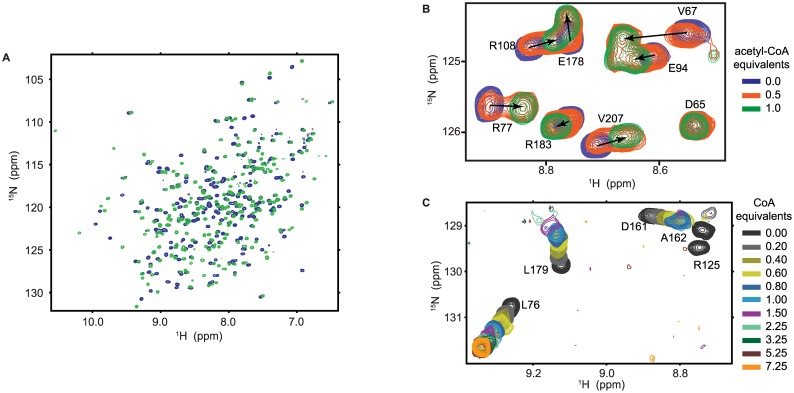
Association of *bm*AANAT3 with acetyl-CoA and CoA followed by NMR. (A) ^15^N-HSQC of *bm*AANAT3 in the free (blue) and acetyl-CoA-bound state (green). The very large number of signals affected by acetyl-CoA indicates that chemical shift perturbations do not solely report cofactor binding at the catalytic funnel, but also induced conformational changes to distal regions of the enzyme. (B) Expanded region of the ^15^N-HSQC of *bm*AANAT3 in the free-state (blue), after the addition of 0.5 (orange) or 1.0 (green) equivalents of acetyl-CoA, showing the concomitant disappearance and appearance of the signals for the free and bound states. (C) Expanded region from the NMR titration course of *bm*AANAT3 with CoA highlighting signals from residues that either do not exhibit any line broadening (L76) or broaden beyond detection at different points of the titration (R125, D161, A162 and L179).

**Fig 2:**
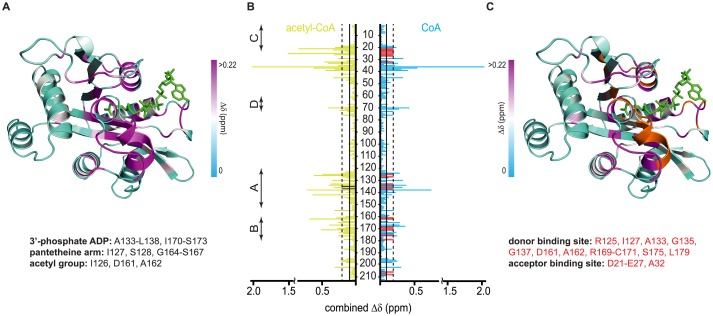
Cofactor-mediated conformational remodeling of *bm*AANAT3 monitored by NMR. (A) acetyl-CoA induced CSPs mapped on the model of *bm*AANAT3. (B) CSPs as a function of primary sequence for the interaction of *bm*AANAT3 with acetyl-CoA (green) and CoA (cyan). CSPs greater than the mean or one SD above the mean are marked by continuous and broken lines, respectively. The conserved motifs of the GNAT superfamily are indicated on the left. Black bars in the acetyl-CoA portion of the plot mark G135 and G137 which are absent in the free state of the enzyme, but tentatively assigned in the bound state. Red bars in the CoA portion of the plot mark residues that become broadened beyond detection in the CoA-bound state. (C) CoA induced CSPs mapped on the model of *bm*AANAT3. In (A) and (C) the white region in the three-color gradient corresponds to the mean CSP, while the magenta region to greater than mean + σ. Residues highlighted in orange in (C) highlight residues that broaden beyond detection in the presence of CoA. The position of acetyl-CoA is highlighted in green, after aligning the *bm*AANAT3 model with the structure of acetyl-CoA-bound DAT. Residues that experience significant CSPs for acetyl-CoA and CoA are listed in (A), while, signals that broaden (beyond detection) upon CoA binding are listed in (C).

To characterize the mode of donor binding, we followed the titration of *bm*AANAT3 with acetyl-CoA and CoA by NMR. Addition of acetyl-CoA to ^15^N-labeled *bm*AANAT3 results in significant chemical shift perturbation (CSP) of a large number of signals (Figs 1A, 2A and 2B), suggesting that acetyl-CoA binding does not affect only residues in the vicinity of the binding site but induces conformational changes to distal regions. Changes in the ^15^N-HSQC spectrum of *bm*AANAT3 as a function of added acetyl-CoA occur on the slow-exchange regime of the NMR timescale, causing progressive disappearance of the signals of the free-state and the concomitant appearance of a new set of signals corresponding to the bound-state (Fig 1B). To discriminate between CSPs reporting direct binding from those reporting induced conformational changes, we took advantage of the highly conserved mode of acetyl-CoA binding to GNAT family members. Thus, we used the structure of DAT (20% identity and 50% similarity) to identify CSPs caused by direct acetyl-CoA binding. As expected, significant CSPs are observed for residues within the conserved motifs A and B (Fig 2A and 2B). Therefore, prominent CSPs that report induced conformational changes outside the acetyl-CoA binding site, span the end of motif C and the downstream sequence prior to motif D, and include amino acids L19-H42 (Fig 2A and 2B). Notably, this region corresponds to a segment of *oa*AANAT’s acceptor binding site which undergoes a loop-to-helix transition upon bisubstrate binding to allow for a high affinity interaction with the acceptor [26, 27].

**Fig 3:**
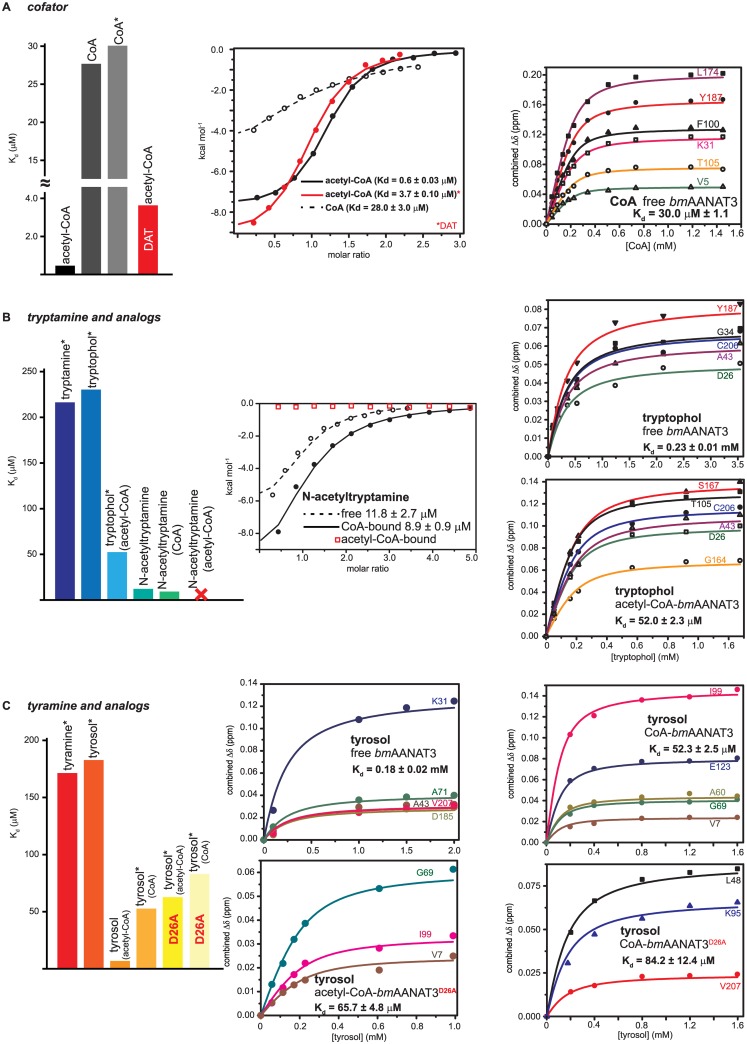
An overview of dissociation constants measured in this study. For those complexes that gave poor *c-values* in the ITC, K_d_ values were determined by NMR (marked by *). A summary of K_d_ values is presented in Table 1. (A) Determination of K_d_ values for the interaction of free *bm*AANAT3 with acetyl-CoA (black) and CoA (gray), and DAT with acetyl-CoA (red). The isotherms presented in the middle panel correspond to interaction of *bm*AANAT3 with acetyl-CoA and CoA (black) and to the interaction of DAT with acetyl-CoA (red). NMR was used to confirm the K_d_ of *bm*AANAT3 for CoA (right panel). (B) Determination of K_d_ values for the interaction of different cofactor-liganded states of *bm*AANAT3 with tryptamine and tryptamine analogs. The presence of acetyl-CoA in the catalytic funnel leads to an increase in the affinity for tryptophol, while an even more substantial increase is observed when an acetylated derivative of tryptamine (N-acetyltryptamine) is utilized. Binding of N-acetyltryptamine to *bm*AANAT3 is not sensitive to the presence of CoA, but it is abolished in the presence of acetyl-CoA. The isotherms presented in the middle panel correspond to the interaction of *bm*AANAT3 with N-acetyltryptamine in the free- or CoA-bound states (black) and in the acetyl-CoA-bound state (red). NMR was used to measure the K_d_ for the interaction of free- and acetyl-CoA-bound *bm*AANAT3 with tryptophol (right panel). (C) Determination of K_d_ values for the interaction of different cofactor-liganded states of *bm*AANAT3 and variants (D26A) with tyramine and tyramine analogs. The presence of CoA in the catalytic funnel leads to an increase in the affinity for tyrosol, while an even more substantial increase is observed in the presence of acetyl-CoA. The D26A mutation renders tyrosol binding insensitive to acetyl-group occupancy of the catalytic center, in a manner that tyrosol affinity of acetyl-CoA-bound *bm*AANAT3^D26A^ is similar to that of CoA-bound *bm*AANAT3^D26A^ or CoA-bound *bm*AANAT3. NMR was used to measure the K_d_ for the interaction of tyrosol with free- or CoA-bound *bm*AANAT3, and for acetyl-CoA- or CoA-bound *bm*AANAT3^D26A^.

To elucidate how conformational changes at this region are linked to the formation of ternary complexes with acceptors, we generated a homology model of *bm*AANAT3 (S1A and S1B Fig). Overall, two major conformational variations are observed between the predicted *bm*AANAT3 structure and the prototypical structure of free *oa*AANAT, both of which occur at the acceptor binding site. First, helix α1 preexists as a long helical element, whereas in *oa*AANAT, helix α1 extends towards its C-terminal end only upon bisubstrate analog binding (S1C Fig). Second, residues 28–33 of *bm*AANAT3, that correspond to the C-terminus of *oa*AANAT’s loop1, form a short helical “plug” on top of the catalytic funnel (S1B Fig). In agreement with the NMR derived secondary structure (S1A Fig), heteronuclear {^1^H}-^15^N NOEs recorded for this region (S1D Fig) indicate a rigid backbone and further support the presence of a preformed long helix α1 and a helical plug in the unliganded state of *bm*AANAT3. These observations explain the significantly higher K_d_ of *oa*AANAT for acetyl-CoA (~242 μM) [35] as compared to that of *bm*AANAT3 (0.66 ± 0.03 μM, Fig 3) and account for the three-fold difference in K_M_ [15, 36]. Hence, the observed CSPs for the L19-H42 segment do not report a loop-to-helix transition, but rather a subtle structural reorganization of helix α1 and the plug over the catalytic funnel. This observation is in line with the proposed ordered mechanism for *bm*AANAT3 and other GNATs, but highlights significant variations within the AANAT class. In this respect, *bm*AANAT3 resembles DAT both in terms of the thermodynamics of acetyl-CoA binding (K_d_ = 3.7 μM, Table 1 and Fig 3) and the steady-state kinetic constants [24, 28, 36].

**Table 1:** Dissociation constants measured for *bm*AANAT3, in μM.

**cofactor**	*acetyl-CoA*	*CoA*				
	0.66 ± 0.03 3.70 ± 0.10^a^	28 ± 3.0				
**tryptamine analogs**	*tryptamine*	*tryptophol*	*Tryptophol (acetyl-CoA)*	*N-acetyltryptamine*	*N-acetyltryptamine (CoA)*	*N-acetyltryptamine (acetyl-CoA)*
	219 ± 8	230 ± 12	52 ± 2.40	11.8 ± 2.7	8.9 ± 0.9	no binding
**tyramine analogs**	*tyramine*	*tyrosol*	*tyrosol (acetyl-CoA)*	*tyrosol (CoA)*	*tyrosol*^b^*(acetyl-CoA)*	*tyrosol*^b^*(CoA)*
	169 ± 17	180 ± 21	6.45 ± 0.52	52.3 ± 2.5	65.7 ± 4.8	84.2 ± 12.4

When *bm*AANAT3 was titrated with an acceptor in the presence of a cofactor (acetyl-Coa or CoA), the cofactor is shown in parenthesis.

^a^ refers to the K_d_ measured for DAT under identical conditions to *bm*AANAT3.

^b^ refers to the K_d_ measured for the *bm*AANAT3 variant D26A.

To further characterize the role of the acetyl group in the progression of the catalytic cycle from an activated acetyl-donor to the CoA product, we investigated the association of *bm*AANAT3 with CoA. Consistent with its higher K_d_ measured by ITC (28.00 ± 3.00 μM, Fig 3A), incremental additions of CoA cause a gradual shift of the signals from the free to the fully saturated state (Fig 1C). Evidently, signals that experience significant CSPs cluster to the same regions as for the titration with acetyl-CoA, and include residues from motif A and motif B (Fig 2B and 2C). This is in agreement with the available structural information for GNATs, where a conserved mode of binding is observed for the two cofactors. Nevertheless, CoA produces distinct conformational changes to *bm*AANAT3 as, for two sets of signals, a change in chemical shift is also associated with significant and incremental signal broadening, leading to complete signal loss (Figs 1C and 2B). Surprisingly, one set maps to the acetyl-CoA binding site and the second to the segment spanning the end of helix α1 and the plug, which partly comprises the acceptor binding site (Fig 2C), indicating that in the presence of CoA the cofactor and acceptor binding sites undergo exchange at a μs-ms timescale. The origin of exchange broadening is not chemical exchange due to CoA binding, since *bm*AANAT3 is fully saturated with the cofactor under these conditions (>99.6%). When ^15^N-HSQC signals from residues that do not experience line broadening but lie in the vicinity of the acetyl-CoA binding site were used to determine the K_d_, there was a very good agreement between NMR and ITC (Fig 3A), confirming saturation with CoA. Therefore broadening is caused by conformational heterogeneity of *bm*AANAT3 in the CoA-bound state, suggesting that the acetyl group of acetyl-CoA (and its absence thereof) modulates the dynamic properties of the enzyme, both locally, throughout the donor binding site, and remotely at the acceptor binding site. Analysis of available crystal structures of GNATs in the presence of either of the two cofactors reveals that, overall, CoA exhibits higher conformational variability as compared to acetyl-CoA. In this respect, the B-factors of CoA atoms are consistently higher than the B-factors of the corresponding atoms of acetyl-CoA (S2 Fig). In addition, there are several structures of CoA-bound GNATs for which poor or no electron density is observed for the adenosine [37–39], pantothenic [40] and β-mercaptoethylamine [37, 40, 41] moieties, or for which multiple conformations are observed, particularly for the pantetheine [42] and β-mercaptoethylamine [43–45] moieties. The inherent mobility observed for CoA segments that are in contact with the enzymes suggests that the CoA-induced conformational heterogeneity observed for *bm*AANAT3 is a common characteristic among GNATs. Thus, although the ΔΔG for CoA and acetyl-CoA binding to *bm*AANAT3 (~-10.0 kJ/mol) indicates a simple additive contribution of the acetyl group to the overall binding affinity of acetyl-CoA as compared to CoA, in the absence of the acetyl group, motions are propagated throughout the cofactor binding site and ultimately to the substrate binding site.

**Fig 4:**
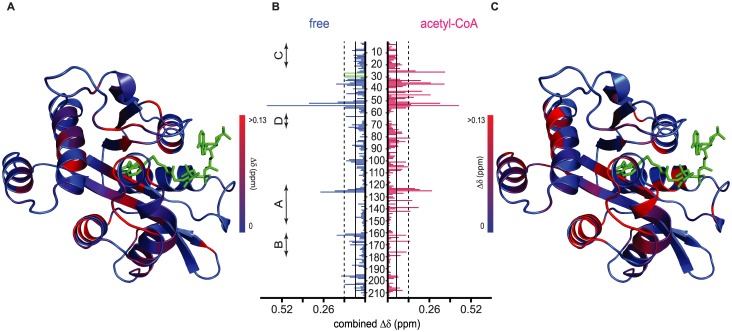
Assembly of *bm*AANAT3 complexes with acceptor substrates monitored by NMR. (A) Mapping of tryptophol induced CSPs to free *bm*AANAT3. The conserved motifs of the GNAT superfamily are indicated on the left. (B) CSPs as a function of primary sequence for the interaction of tryptophol with free- (blue) and acetyl-CoA-bound *bm*AANAT3 (red). CSPs greater than the mean or one SD above the mean are marked by continuous and broken lines, respectively. Green, open bars in the free-state mark residues that disappear in the substrate-bound state. (C) Mapping of tryptophol induced CSPs to acetyl-CoA bound *bm*AANAT3. The bisubstrate, CoA-S-acetyltryptamine (green), was modelled by aligning *bm*AANAT3 model to the structure of *oa*AANAT.

The conformational heterogeneity of the acceptor binding site in the CoA-bound form suggests that acetyl-group occupancy in the catalytic funnel may control the sequential mechanism. To test this hypothesis, we characterized binary and ternary complexes of *bm*AANAT3 with acceptor substrates. To prevent acetyl group transfer in ternary complexes, we used tyrosol and tryptophol as model non-acceptor analogs of tyramine and tryptamine, respectively [15]. Both ligands give poor ITC isotherms with low c-values and thus NMR titrations were used to determine K_d_ values for binary complexes (Fig 3B and 3C). In agreement with an ordered kinetic mechanism, saturation occurs only in the presence of high acceptor excess (K_d_ values of 180 ± 21 and 230 ± 12 μM for tyrosol and tryptophol, respectively), indicating a weak acceptor-*bm*AANAT3 interaction. Mapping the observed CSPs on the model of *bm*AANAT3 (Fig 4) reveals that the most prominent changes occur at the following structural elements (i) the β-sheet, at a position of the catalytic funnel that coincides with the tryptamine moiety of the bisubstrate in the crystal structure of *oa*AANAT, (ii) the plug and the downstream loop, (iii) helix α2, (iv) the insect-specific helical insert and (v) the loop connecting the last two β-strands on top of the catalytic funnel. In the homology model of *bm*AANAT3 generated using the crystal structure of *aa*AANAT2, a large number of hydrophobic sidechains from these regions protrude towards the catalytic funnel forming a hydrophobic cage, which presumably encapsulates the arylalkyl acceptor (S3 Fig). A similar cluster of hydrophobic residues is also observed in the crystal structures of DAT in both the free- and acetyl-CoA-bound states [28]. In addition, a set of residues within the plug (E27, L29 and N30) exhibit significant line broadening or disappear at near saturating substrate concentrations (Fig 4). Thus, although the acceptor interacts weakly with the hydrophobic part of the catalytic funnel even in the context of binary acceptor complexes, the dynamic nature of the plug does not allow for a high affinity interaction.

On the other hand, in the presence of acetyl-CoA the interaction with acceptors is significantly enhanced (K_d_ values of 6.45 ± 0.52 and 52 ± 2.40 μM, for tyrosol and tryptophol, respectively, Fig 3B and 3C). To identify the molecular origin of this strong, positive binding cooperativity (ΔΔG ~ -8 kJ/mol), we looked for differences in the NMR titrations of substrates in free and acetyl-CoA-bound *bm*AANAT3. The CSP patterns obtained from the two titrations are very similar (Fig 4), suggesting that the mode of substrate binding at the two states is similar. However, in the presence of acetyl-CoA, residues at the C-terminal end of helix α1 that either contribute directly to the substrate binding site (F22) or lie in the vicinity of the 3’-phosphate ADP moiety binding site (A25, D26), experience significantly higher chemical shift changes. Furthermore, the residues from the plug for which we have available assignment in the acetyl-CoA-bound state, do not show any line broadening. These results suggest that acetyl-CoA binding selectively stabilizes a conformation of the plug which is competent for a strong interaction with the substrate.

The differential effect of CoA and acetyl-CoA binding on the conformational properties of the acceptor binding site, implies that acetyl group occupancy of the catalytic cavity may act as an effector that modulates the affinity of the enzyme for acceptors during the ordered catalytic cycle. To test this hypothesis we studied the interaction of acceptors with CoA-bound *bm*AANAT3. Indeed, the affinity of tyrosol is significantly reduced as compared to the acetyl-CoA-bound state of *bm*AANAT3 (K_d_ of 52.3 ± 2.5 μM), although it is still higher than for the free enzyme. Hence, the extent of the binding cooperativity is highly dependent on the presence of the acetyl group on the cofactor and binding of CoA does not shift *bm*AANAT3 to an acceptor binding competent state to the same extent as acetyl-CoA does. Next, we utilized N-acetyltryptamine as an acetylated acceptor to test binding to free *bm*AANAT3 and to a binary complex with CoA. N-acetyltryptamine shows a 20-fold increase in affinity for the free protein as compared to the non-acetylated substrate (K_d_ = 11.8 ± 2.7 μM), which is only marginally improved in the presence of CoA (K_d_ = 8.9 ± 0.9 μM) (Fig 3). It must be noted that this large difference in K_d_ is not due to an erroneous comparison between the alcohol mimic and the natural amine, as the affinity of tyramine and tryptamine is comparable to that tyrosol and tryptophol for free *bm*AANAT3 (Fig 3). Thus, acetyl group occupancy of the catalytic site has a profound effect on the binding properties of the acceptor binding site even when the acetyl group is carried by the acceptor. Importantly, the position of the acetyl group of the acetylated acceptor is mutually exclusive to the position of the acetyl group of acetyl-CoA, as N-acetyltryptamine does not bind to *bm*AANAT3-acetyl-CoA (Fig 3B). This is expected to provide a mechanism for productive dissociation of the acetylated acceptor upon acetyl-CoA binding during the progression to a new catalytic cycle. In support of this hypothesis, comparison of available GNAT structures in the presence of acetyl-CoA and bisubstrates or acetylated acceptors, shows that the acetyl group occupies the same space and in some cases with a conserved hydrogen bond network [46–50].

As the most significant difference between the NMR titrations of *bm*AANAT3 with acetyl-CoA and CoA was the conformational heterogeneity of the C-terminus of helix α1 and the plug, we hypothesized that residues at the interface between this region and the P-loop encompass the molecular switch that couples acetyl group occupancy to the acceptor binding site. Comparison of the structure of DAT in the free and acetyl-CoA-bound state reveals that a salt-bridge between D46 and R153 is only formed in the presence of acetyl-CoA, while a salt bridge from residues of the same structural elements of *oa*AANAT (E54 and R131) is observed in the presence of a bisubstrate, but not in the free-state. To test our hypothesis, we mutated the corresponding residue, D26, of *bm*AANAT3 to alanine (S4 Fig). Our rationale was that disruption of a potential interaction with R134 from the P-loop in the acetyl-CoA-bound state would affect the substrate binding properties. Indeed, tyrosol binding to acetyl-CoA-bound *bm*AANAT3^D26A^, shows a 10-fold drop in affinity (K_d_ = 65.7 ± 4.8 μM), to the level of CoA-bound *bm*AANAT3 or CoA-bound *bm*AANAT3^D26A^ (Kd = 84.2 ± 12.4 μM) (Fig 3C). Hence, this salt bridge comprises the structural switch that mediates the observed positive cooperativity by sensing acetyl group occupancy at the catalytic site, since when broken, acetyl group occupancy of the catalytic funnel is not relayed to the substrate binding site. This observation is also in agreement with the 20-fold higher *K*_m,app_ observed for DAT^R153A^ for tyramine as compared to wild type DAT [24].

## Conclusions

In summary, we find that the sequential progression between binary and ternary complexes formed during the catalytic cycle of an N-acetyltransferase is coordinated by the acetyl group occupancy in the catalytic funnel. When carried by acetyl-CoA, the acetyl group shifts the conformational ensemble of the acceptor binding site to a high affinity competent state, while when carried by the acceptor it allows for displacement of the acetylated acceptor upon acetyl-CoA binding. Our NMR data also show that the C-terminus of helix α1 of *bm*AANAT3 preexists in a helical conformation in the free-state of the enzyme and thus allows for a high affinity interaction with the cofactor, acetyl-CoA. Similar observations were made for the free-states of DAT and *aa*AANAT2, suggesting that this is a general mechanism for insect acetyltransferases [22, 28]. However, the highly conserved sequential mechanism observed for other GNATs suggests that this model may apply to other members of the superfamily.

## Supporting information

S1 Fig Structural properties of *bm*AANAT3. (A) Primary sequence alignment of *bm*AANAT3 and of the template, *aa*AANAT2, generated using Jalview and color-coded according to amino acid properties. Invariant residues are marked with an asterisk. A comparison between the secondary structure elements predicted by homology modeling (noted as *predicted SS*) to those determined by analysis of H^N^, N, C_α_, C_β_ and C’ backbone chemical shifts of *bm*AANAT3 (noted as *NMR SS*) is shown under the alignment. Strands and helices are represented by arrows and cylinders, respectively, and named according to the GNAT nomenclature. Conserved motifs of the GNAT superfamily and amino acid numbering is shown above the alignment. (B) Orthogonal views of the homology model of *bm*AANAT3, where the conserved GNAT family fold is colored in red. The insect specific *helical insert* and the helical *plug*, are shown in cyan. The bisubstrate analog CoA-S-Acetyltryptamine (shown in green) was used to illustrate the position of acetyl-donor and -acceptor ligands in the catalytic funnel, after structural alignment of the model with the crystal structure of the binary complex of *oa*AANAT with a bisubstrate analog (PDBID: 1CJW). (C) Overlay of the *bm*AANAT3’s homology model (colored as in panel B) to the structures of *oa*AANAT in the free (yellow) and bisubstrate-bound state (blue). The bisubstrate analog is shown in green and loop1, which undergoes a disorder-to-helix transition upon bisubstrate binding is shown in gray. In the extended conformation, loop1 partially occupies binding of acetyl-CoA, while in the helical conformation it completes the substrate binding site. These differences may account for the higher K_d_ of *oa*AANAT for acetyl-CoA as well as for the higher K_M_ observed, compared to *bm*AANAT3. (D) {^1^H}-^15^N heteronuclear NOEs, which are sensitive to ps-ns timescale dynamics of backbone atoms, reveal that the plug region (colored in magenta) acquires a helical conformation in solution.
(EPS)

S2 Fig Correlation between acetyl-CoA and CoA B-factors. A comparison between the B-Factors of acetyl-CoA and CoA atoms obtained from those GNAT crystal structures that have been determined with both acetyl-CoA and CoA. The PDB IDs for each set of structures is provided.
(EPS)

S3 Fig Proposed acceptor substrate binding cavity. Two orthogonal views of the acceptor substrate binding cavity of the modelled *bm*AANAT3 structure. The tryptamine and CoA moieties of the bisubstrate analog CoA-S-acetyltryptamine are colored pink and yellow respectively, while hydrophobic residues that exhibit significant chemical shift perturbation are shown as green spheres.
(EPS)

S4 Fig Acetyl-group donor and acceptor coupling switch. Cartoon diagram of *bm*AANAT3 model highlighting the salt bridge between D26 located at the end of helix α1 and R134 located at the P-loop.
(EPS)
